# Minocycline reduces neuroinflammation but does not ameliorate neuron loss in a mouse model of neurodegeneration

**DOI:** 10.1038/srep10535

**Published:** 2015-05-22

**Authors:** Shanshan Cheng, Jinxing Hou, Chen Zhang, Congyu Xu, Long Wang, Xiaoxia Zou, Huahong Yu, Yun Shi, Zhenyu Yin, Guiquan Chen

**Affiliations:** 1 Model Animal Research Center, MOE Key Laboratory of Model Animal for Disease Study, Nanjing University, 12 Xuefu Road, Nanjing, Jiangsu Province, 210061, China; 2 School of Life Sciences, Nanchang University, Nanchang, Jiangxi Province, 330031, China; 3 Department of Geriatric, Nanjing Drum Tower Hospital, Nanjing University Medical School, 321 Zhongshan Avenue, Nanjing, Jiangsu Province, 210008, China

## Abstract

Minocycline is a broad-spectrum tetracycline antibiotic. A number of preclinical studies have shown that minocycline exhibits neuroprotective effects in various animal models of neurological diseases. However, it remained unknown whether minocycline is effective to prevent neuron loss. To systematically evaluate its effects, minocycline was used to treat *Dicer* conditional knockout (cKO) mice which display age-related neuron loss. The drug was given to mutant mice prior to the occurrence of neuroinflammation and neurodegeneration, and the treatment had lasted 2 months. Levels of inflammation markers, including glial fibrillary acidic protein (GFAP), ionized calcium-binding adapter molecule1 (Iba1) and interleukin6 (IL6), were significantly reduced in minocycline-treated *Dicer* cKO mice. In contrast, levels of neuronal markers and the total number of apoptotic cells in *Dicer* cKO mice were not affected by the drug. In summary, inhibition of neuroinflammation by minocycline is insufficient to prevent neuron loss and apoptosis.

Neurodegeneration occurs in a group of diseases displaying progressive loss of neurons from the nervous system^1^. Based on its distinct symptoms and brain areas affected, neurodegenerative disease (ND) is classified as Alzheimer’s disease (AD), Parkinson’s disease (PD), Huntington’s disease (HD), frontotemporal dementia (FTD) and amyotrophic lateral sclerosis (ALS)^2^. Other important features of ND include neuroinflammation and abnormal protein assemblies^3^. It has been shown that neuron loss and tau phosphorylation are increased in parallel with the severity of dementia, and that neuron loss contributes directly to cognitive impairment in AD^4^.

In the central nervous system (CNS), neuroinflammation is mediated by microglia and astrocytes, which produce inflammatory cytokines, reactive oxygen species, and other toxic materials^5^. In AD, there are increased levels of inflammatory cytokines and chemokines such as IL1β^6^, IL6^7^, IL8^8^, tumor necrosis factor-α (TNFα)^9^, macrophage inflammatory protein1β (MIP1β)^10^ and monocyte chemoattractant protein1 (MCP1)^11^. Neuroinflammation, including reactive astrocytes and activated microglia, is widely seen in AD^12^ and correlates with cognitive decline and brain atrophy^8^. Accumulating evidence has indicated that anti-inflammatory agents are protective for AD^13^. Neuroinflammation was reported to take place prior to overt neuron loss in various animal models displaying age-related neuron loss^14^,^15^,^16^. Therefore, neuroinflammation is an early event of neurodegeneration and may play a critical role in the disease progression. Indeed, neuroinflammation is believed to be a driving force for neurodegeneration^17^, raising the possibility that the early use of anti-inflammation drugs may prevent neuron loss.

Minocycline is a broad-spectrum tetracycline antibiotic, and can readily cross the blood-brain barrier to exert beneficial effects such as anti-inflammatory, anti-apoptotic and neuroprotective in animal models of neurological diseases^18^,^19^. Several studies have demonstrated that minocycline inhibits neuroinflammation and neuron death in mouse models of AD with amyloid plaques^20^,^21^, ALS^22^, HD^23^, PD^24^, Down’s syndrome^25^, and stroke^26^,^27^. Minocycline prevents Aβ deposition and improves cognitive deficits in amyloid precursor protein (APP) transgenic (Tg) models of AD^21^,^28^,^29^, and it also inhibits tau phosphorylation and prevents aggregation of insoluble tau likely through inhibiting caspase3 activation in a Tg mouse model of tauopathy^30^,^31^. The evidence above indicates that minocycline is effective to reduce plaque and tangle pathology. Since it has been unknown whether minocycline could be used as a valuable anti-neurodegeneration drug for ND, it is of great importance to evaluate its preclinical efficacy using appropriate neurodegenerative mouse models.

Impaired microRNA (miR or miRNA) network due to loss of endoribonuclease Dicer affects early cortical development and morphogenesis^32^,^33^,^34^. It has been shown that conditional deletion of Dicer in different brain areas causes age-related neurodegeneration^35^,^36^,^37^. In this study, 2 months old *Dicer* cKO mice exhibiting neither neuroinflammation nor neuron loss had received minocycline treatment for 2 months. We found that neuroinflammation was effectively inhibited, and that neuron loss and apoptosis were not ameliorated.

## Results

### *Dicer* cKO mice at the age of 2 months showed normal brain morphology

To generate neuron-specific *Dicer* cKO mice, we bred floxed *Dicer* (*Dicer*^*f/f*^)^38^ to the T29-1 line of *CaMKIIα-Cre*^39^,^40^. Mice with the genotype of *Dicer*^*f/f*^*;CaMKIIα-Cre* were designated as *Dicer* cKO. In the T29-1 line, the expression pattern of Cre recombinase has been fully characterized^40^. The Cre expression starts from about 1.5-2 months in forebrain excitatory neurons of adult mice^40^. Unlike two previously published *Dicer* cKO lines^32^,^37^, the line generated in this study did not show abnormal postnatal death^16^.

**Figure 1:**
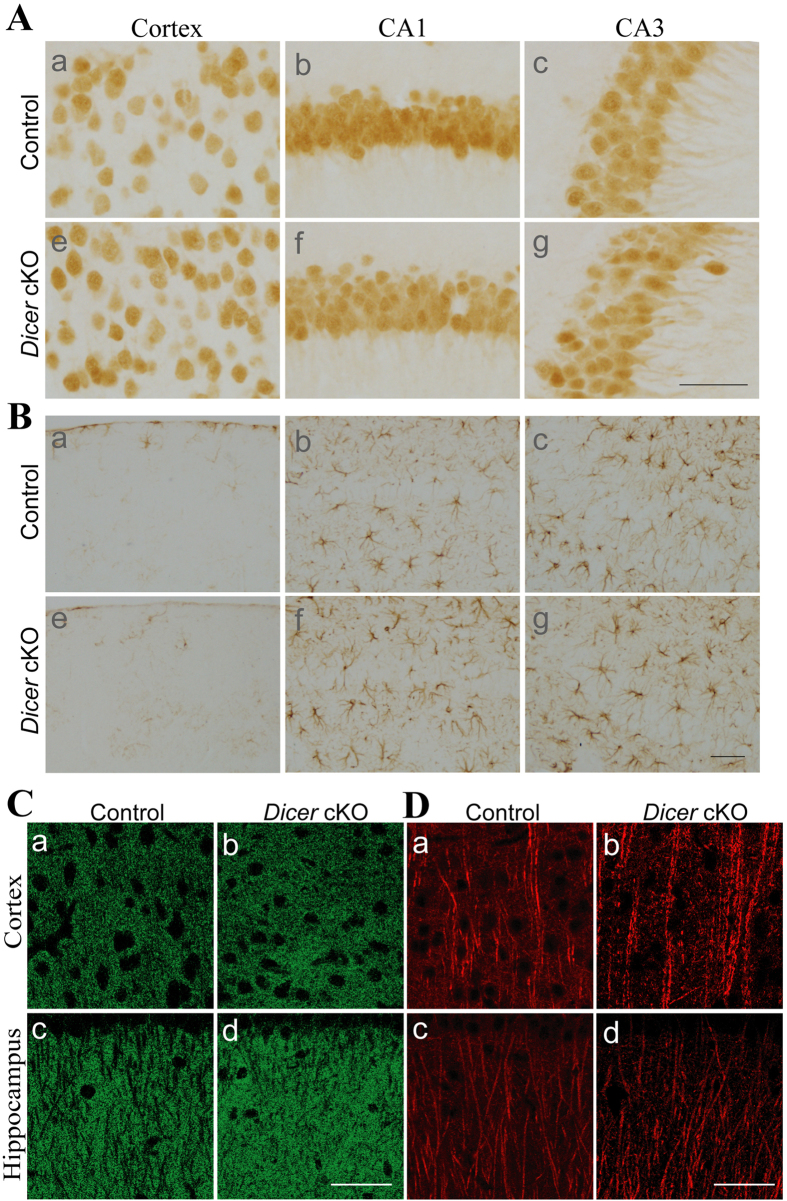
No morphological changes in *Dicer* cKO mice at the age of 2 months. **** (**A**)Immunohistochemistry for NeuN. The number of NeuN+cells in the brain of *Dicer* cKO mice (e-g) was not different from that in control mice (**a**–**c**). (**B**) Immunohistochemistry for GFAP. The number of GFAP+cells in *Dicer* cKO (e-g) and control mice (**a**–**c**) was comparable. (**C**) Immunohistochemistry for SVP38. SVP38 immuno-reactivity in *Dicer* cKO mice (b,d) was not different from that in control mice (a,c). (**D**) Immunohistochemistry for MAP2. MAP2 antibody was used to label dendritic structure. MAP2 immuno-reactivity showed no difference between *Dicer* cKO and control mice. Scale bar = 50μm.

We first examined whether young (2 months old) *Dicer* cKO displayed neuron loss. We conducted Nissl staining and observed no detectable cortical atrophy (data not shown). We then performed immunohistochemistry (IHC) for NeuN (a marker for mature neuron) and GFAP (a marker for astrocyte). There were no differences in NeuN immuno-reactivity and the number of NeuN positive (+) cells in the cortex, hippocampal CA1 and hippocampal CA3 areas of control and *Dicer* cKO mice (Fig. 1A). There was no change in GFAP immuno-reactivity as well (Fig. 1B). Western analyses confirmed no changes in levels of NeuN (data not shown) and GFAP (data not shown) in *Dicer* cKO at this age. To determine whether synaptic morphology was affected, we examined levels of synaptophysin (SVP38) and post-synaptic density 95 (PSD95), markers for pre- and post-synaptic components, respectively. No significant difference was found in levels of SVP38 (control = 100% ± 1.1%, cKO = 105.1% ± 10.7%) and PSD95 (control = 100% ± 0.6%, cKO = 100.1% ± 8.7%) in *Dicer* cKO mice (ps > 0.3). IHC for SVP38 (Fig. 1C) revealed no change in synaptic morphology at 2 months. IHC for microtubule-associated protein2 (MAP2) (Fig. 1D) further showed no change in dendrite morphology.

To determine at which age *Dicer* cKO mice start to exhibit neuroinflammation and neuron loss, we conducted IHC for GFAP and NeuN using mice at the age of 2 months, 11 and 13 weeks. We observed age-dependent increase in GFAP immuno-reactivity. Increased number of GFAP+cells was observed in *Dicer* cKO at 11 weeks, suggesting early astrocytosis (Fig. S1A). More severe astrocytosis was found in *Dicer* cKO at 13 weeks (Fig. S1A). In contrast, no detectable change in NeuN immuno-reactivity was found in the cortex and the hippocampus of *Dicer* cKOs at 2 months (Fig. 1A), 11 and 13 weeks (Fig. S1B). Thus, the occurrence of neuroinflammation precedes overt neuron loss in this mouse model. The age of 2 months was chosen as the starting point for the drug treatment.

### Minocycline reduced neuroinflammation in *Dicer* cKO mice

**Figure 2:**
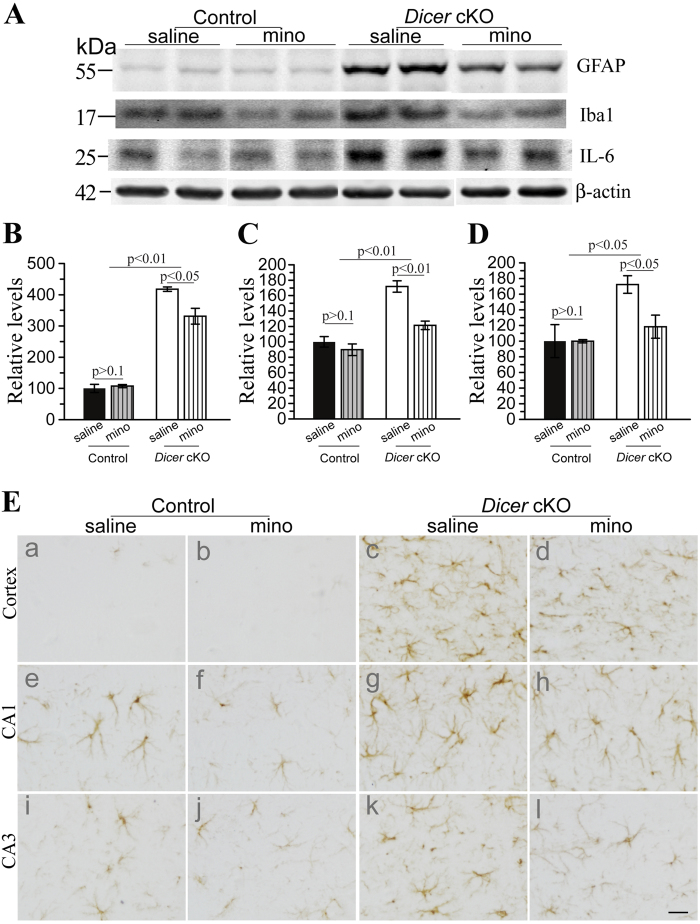
Minocycline reduced inflammatory responses in *Dicer* cKO mice. **** (**A**) Western blotting for GFAP, Iba1 and IL6. β–actin served as the internal control. Representative WB bands for 4 groups of mice were shown. (**B**) Quantitative results for GFAP. There was significant difference in GFAP levels between control and *Dicer* cKO mice. GFAP levels in minocyline-treated cKOs significantly differed from those in saline-treated cKOs. GFAP levels in minocyline-treated control did not differ from those in saline-treated control. (**C**) Quantitative results for Iba1. Levels of Iba1 in minocyline-treated cKOs were significantly reduced as compared to saline-treated cKOs. The two control groups did not differ (p > 0.1). (**D**) Quantitative results for IL6. Levels of IL6 in minocyline-treated cKOs were significantly lower than those in saline-treated cKOs, and were not different from those in control mice. (**E**) Immunohistochemistry for GFAP. Abundant GFAP+cells were observed in the *Dicer* cKO mice. Minocyline-treated cKOs (d,h,l) showed less number of GFAP+cells than saline-treated cKOs (c,g,k) did. There was no difference in the number of GFAP+cells between minocyline- (b,f,j) and saline-treated control (a,e,i) mice. Scale bar = 20μm. Raw Western blotting images for GFAP, Iba1 and IL6 were shown in Supplementary Figures 2-3.

Following a 2-month period of minocycline treatment, several neuroinflammation markers were examined. We performed biochemical analysis and found that GFAP levels were massively increased in *Dicer* cKOs aged at 4 months (Fig. 2A). GFAP levels in the two control groups were not different (saline = 100% ± 12.9%, minocycline = 108% ± 4.9%, p > 0.1). Interestingly, GFAP levels in minocyline-treated cKOs were significantly lower than those in saline-treated cKOs (Fig. 2B: saline = 417.9% ± 7.0%, minocycline = 331.4% ± 25.3%, p < 0.05). Faint immuno-reactivity for GFAP was seen in brains of control groups (Fig. 2E-a,b,e,f,i,j). Abundant GFAP+cells were detected in the cortex and the hippocampus of minocyline- and saline-treated cKO mice (Fig. 2E-c,d,g,h,k,l). GFAP immuno-reactivity was reduced in minocyline-treated cKOs (Fig. 2E-d,h,l), as compared to saline-treated cKOs (Fig. 2E-c,g,k).

To examine whether minocycline affected microglial activation, we performed Western blotting for Iba1, a marker for microglia. We found increased intensities for the Iba1 band in 4-month-old *Dicer* cKOs (Fig. 2A). First, there was no significant difference in Iba1 levels in the two control groups (Fig. 2C: saline = 100% ± 6.9%, minocycline = 89.7% ± 7.6%, p > 0.1). Second, Iba1 levels were significantly reduced in minocyline-treated cKOs than in saline-treated cKOs (Fig. 2C: saline = 171.9% ± 7.4%, minocycline = 121.5% ± 5.5%, p < 0.01), suggesting that microglial activation was inhibited. Since elevated levels of IL6, an inflammatory cytokine, were seen in neurodegenerative brains^7^,^41^, we conducted Western analysis (Fig. 2A). IL6 levels in minocyline-treated cKOs were significantly lower than those in saline-treated cKOs (Fig. 2D: saline = 172.3% ± 11.2%, minocycline = 118.4% ± 14.8%, p < 0.05). Moreover, IL6 levels in minocyline-treated cKOs did not differ from those in control mice (p > 0.1). Overall, 2-month minocycline treatment effectively inhibited neuroinflammation.

### Minocycline did not ameliorate neuron loss in *Dicer* cKO mice

**Figure 3:**
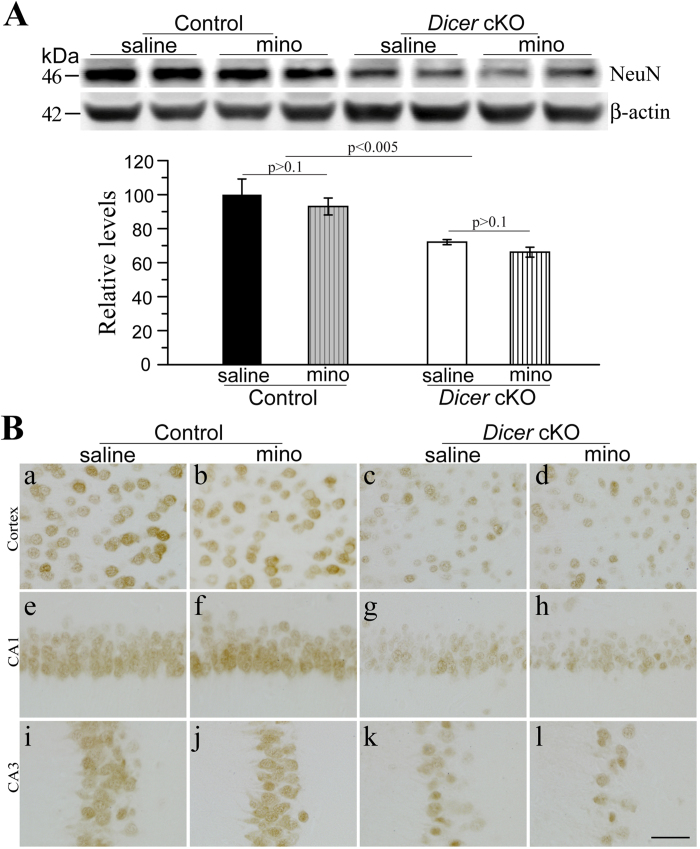
Minocycline did not ameliorate neuron loss in *Dicer* cKO mice. **** (**A**) Western blotting for NeuN using cortical lysates. β–actin served as the internal control. In the bar graph, there was significant difference in NeuN levels between control and cKOs. NeuN levels were not different between saline- and minocycline- treated *Dicer* cKOs. (**B**) Immunohistochemistry for NeuN. *Dicer* cKO mice (c,d,g,h,k,l) exhibited less number of NeuN+cells than control animals (a,b,e,f,i,j) did. However, there was no difference in the number of NeuN+cells between minocyline- and saline-treated *Dicer* cKOs. Scale bar = 40μm. Raw Western blotting images for NeuN were shown in Supplementary Figure 4.

To determine whether minocycline affected neurodegeneration in *Dicer* cKO mice, NeuN^37^,^42^ was examined using biochemical and morphological methods. Compared to control mice, *Dicer* cKOs showed significantly decreased NeuN levels at 4 months (Fig. 3A). Quantitative data showed about a 30% reduction on total NeuN levels in saline-treated *Dicer* cKOs, suggesting significant neuron loss. Indeed, analysis of variance (ANOVA) revealed a highly significant main genotype effect (p < 0.005) but no drug effect (p > 0.3). NeuN levels in minocyline-treated controls were not different from those in saline-treated controls (saline = 100% ± 10.1%, minocycline = 93% ± 5.0%, p > 0.1). No significant difference in NeuN levels was found between minocyline- and saline-treated cKOs (Fig. 3A: saline = 72.1% ± 1.5%, minocycline = 66.2% ± 2.9%, p > 0.1). These results suggest that there was no rescue effect by minocycline.

Furthermore, IHC for NeuN was conducted. Abundant NeuN+cells were found in control brains (Fig. 3B-a,b,e,f,i,j), and there was no difference between minocyline- and saline-treated control mice. However, much less number of NeuN+cells was found in the cortex and the hippocampus of *Dicer* cKO mice as compared to controls. NeuN immuno-reactivity was not different between minocyline- and saline-treated cKO mice (Fig. 3B-c,d,g,h,k,l). Overall, 2-month minocycline treatment did not ameliorate neuron loss.

### Minocycline did not attenuate synaptic loss in *Dicer* cKO mice

**Figure 4:**
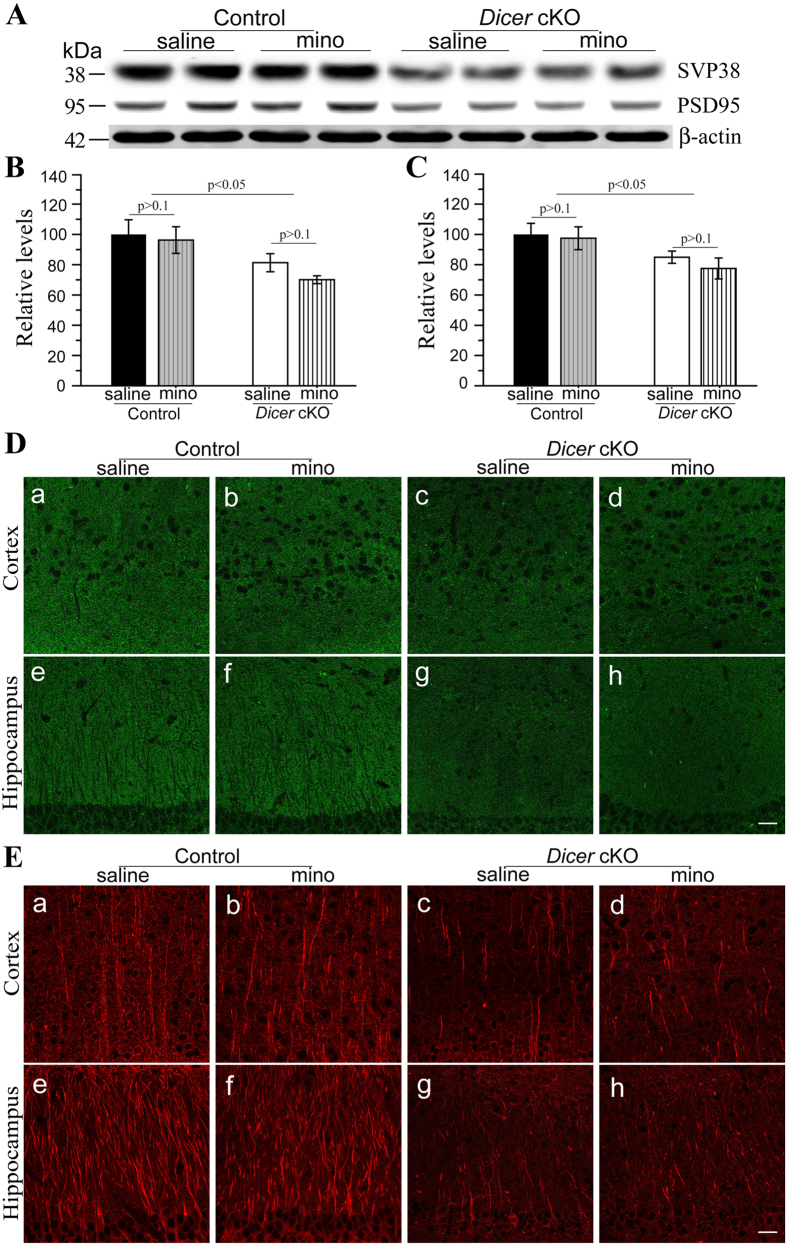
Minocycline did not rescue synaptic and dendritic loss in *Dicer* cKO mice. **** (**A**) Western blotting for SVP38 and PSD95. Cortical samples of 4 groups of mice were used. β–actin served as the internal control. (**B**) Quantitative results for SVP38. There was significant difference in SVP38 levels between control and cKO mice (p < 0.05). There was no difference in SVP38 levels between minocyline- and saline-treated *Dicer* cKO mice (p > 0.1). (**C**)Quantitative results for PSD95. There was significant difference in PSD95 levels between control and cKO mice (p < 0.05). However, there was no difference in PSD95 levels between minocyline- and saline- treated *Dicer* cKO mice (p > 0.1). (**D**) Immunohistochemistry for SVP38. Significantly reduced SVP38 immuno-reactivity was found in *Dicer* cKO mice (c,d,g,h), as compared to control mice (a,b,e,f). There was no difference in SVP38 immuno-reactivity between minocyline- and saline-treated cKO mice. (**E**) Immunohistochemistry for MAP2. Compared to control mice (a,b,e,f), *Dicer* cKO (c,d,g,h) exhibited significantly decreased MAP2 immuno-reactivity in the cortex and the hippocampus. However, there was no difference in MAP2 immuno-reactivity between minocyline- and saline-treated *Dicer* cKO mice. Scale bar = 25μm. Raw Western blotting images for SVP38 and PSD95 were shown in Supplementary Figures 5-6.

To examine the effect of minocycline on synapses, we conducted Western blotting for SVP38 and PSD95. In Fig. 4A–C, ANOVA revealed significant genotype effects (ps < 0.05) but no drug effects (ps > 0.3) on levels of SVP38 and PSD95, suggesting reduced levels of pre- and post-synaptic components in *Dicer* cKOs. However, levels of SVP38 in minocyline- and saline-treated *Dicer* cKOs did not differ (Fig. 4B: saline = 81.3% ± 6.0%, minocycline = 70.1% ± 2.6%, p > 0.1). PSD95 levels in minocyline- and saline-treated cKOs were not different (Fig. 4C: saline = 85.0% ± 4.0%, minocycline = 77.5% ± 6.9%,p > 0.1). While immuno-reactivity of SVP38 was strong in the cortex and the hippocampus of control mice (Fig. 4D-a,b,e,f), it was quite weak in *Dicer* cKO mice (Fig. 4D-c,d,g,h). There was no significant difference between the two cKO groups. Hence, minocycline did not prevent synaptic loss caused by conditional inactivation of Dicer.

To examine whether minocycline affects dendrite morphology, we carried out IHC for MAP2. Strong MAP2 immuno-reactivity was seen in control groups (Fig. 4E-a,b,e,f). The integrity of MAP2-labeled dendrites was largely disrupted in *Dicer* cKO mice (Fig. 4E-c,d,g,h). Moreover, there was no detectable improvement on MAP2 immuno-reactivity or MAP2-labelled dendritic structure in *Dicer* cKOs after the minocycline treatment. Overall, minocycline did not prevent synaptic and dendritic loss.

### Minocycline did not inhibit apoptosis in *Dicer* cKO mice

**Figure 5:**
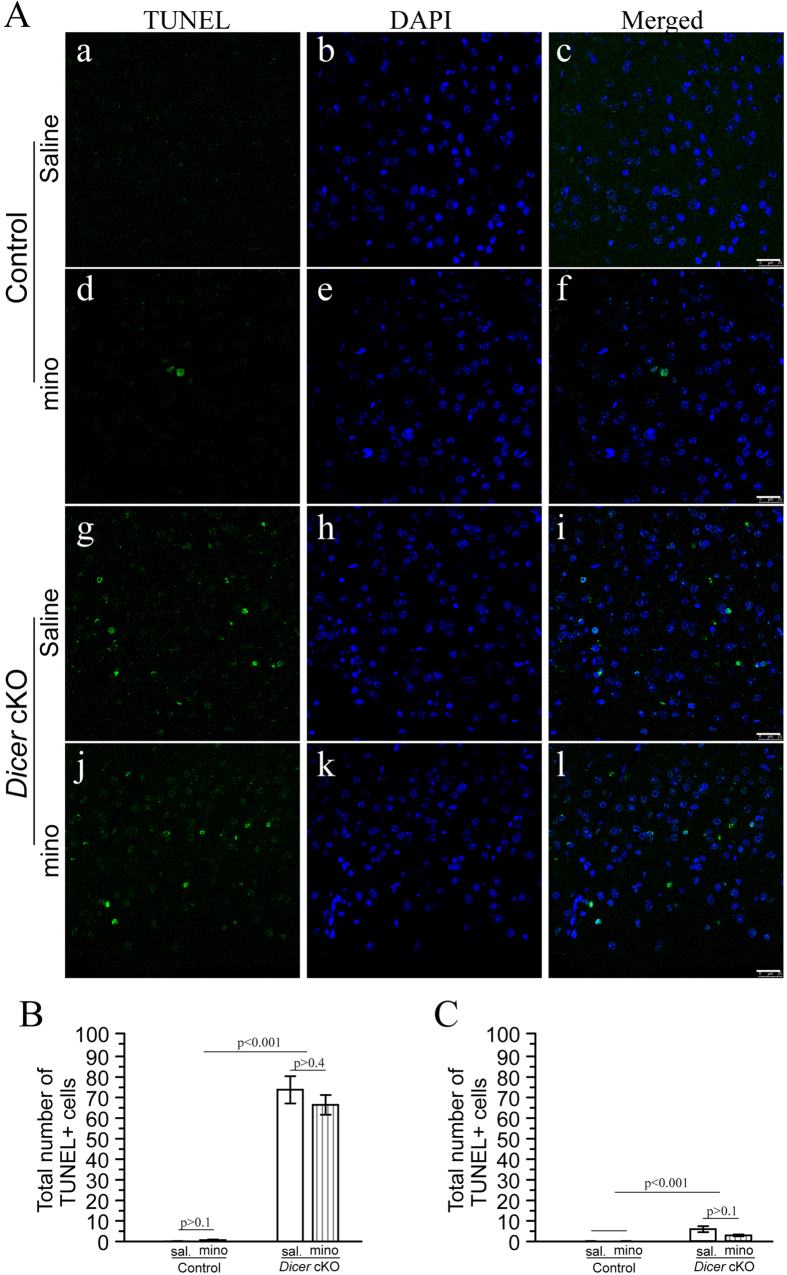
Minocycline did not inhibit apoptosis in *Dicer* cKO mice. **** (**A**) TUNEL and DAPI staining in the cortex for *Dicer* cKO mice. TUNEL+(a,d,g,j) cells were shown in green and DAPI+(b,e,h,k) cells in blue (a-f: control; g-l: cKO). TUNEL+cells were hardly found in control mice (a,d), but were readily seen in *Dicer* cKO (g,j) mice. Scale bar = 25 μm. (**B**) Quantitative results on the average number of TUNEL+cells in the cortex per section. *Dicer* cKO mice exhibited>60 TUNEL+cells in average, and were significantly different from controls (p < 0.001). Minocycline did not change the total number of TUNEL+cells. (**C**) Quantitative results on the average number of TUNEL+cells in the hippocampus per section. *Dicer* cKOs showed significantly more number of TUNEL+cells than controls did (p < 0.001).

We previously reported that *Dicer* cKO mice display increased apoptosis in the cortex^16^. To determine whether minocycline affects apoptosis, we performed the terminal deoxynucleotidyl transferase-mediated dUTP-biotin nick end-labeling (TUNEL) assay. First, abundant TUNEL+cells were observed in 4-month-old *Dicer* cKO mice (Fig. 5A-g,j). Second, we counted total number of TUNEL+cells using a stereological method. The average number of TUNEL+cells per section for the cortex in each group was plotted (Fig. 5B). Third, ANOVA revealed a highly significant main genotype effect (p < 0.001). A total number of >60 TUNEL+cells in the cortex were found in each section of the cKO groups (Fig. 5A:g–l). In contrast, TUNEL+cells were hardly seen in each section of the control groups (Fig. 5A:a–f). Fourth, there was no significant difference in the total number of TUNEL+cells in the cortex of minocycline- and saline-treated cKO mice (Fig. 5B: minocycline = 66.4 ± 4.8, saline = 73.8 ± 6.6, p > 0.4). Fifth, there was also no significant difference in the total number of TUNEL+cells in the hippocampus (Fig. 5C: p > 0.1). Therefore, apoptosis was not inhibited by minocycline.

### Minocycline did not reduce tau hyperphosphorylation in *Dicer* cKO mice

**Figure 6:**
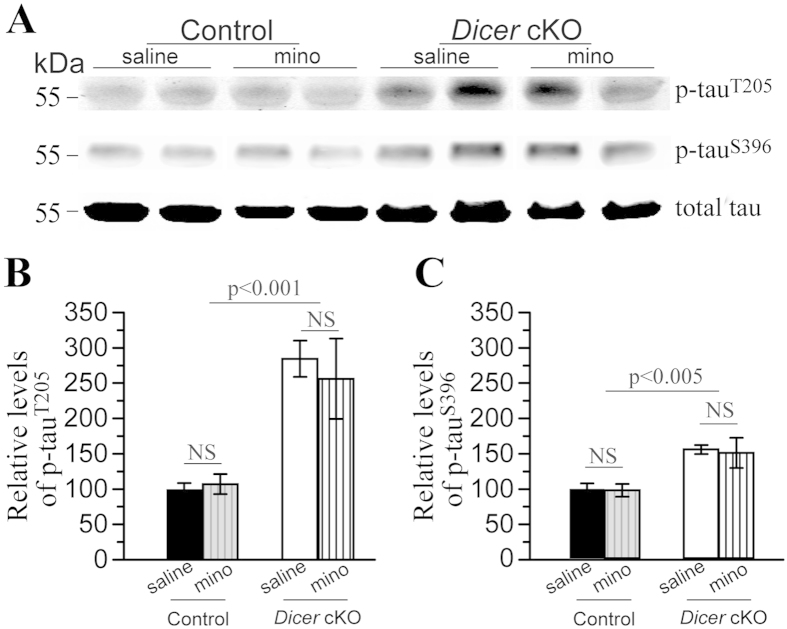
Minocycline did not reduce tau hyperphosphorylation in *Dicer* cKO mice. **** (**A**) Western blotting for p-tau. Representative bands for p-tau^T205^, p-tau^S396^ and total tau were shown. (**B**) Quantitative results for p-tau^T205^. Relative levels of p-tau^T205^ to total tau were plotted for four groups of mice. There was significant increase in p-tau^T205^ levels in *Dicer* cKO mic*e*, as compared to control animals. There was no difference between minocyline- and saline-treated *Dicer* cKO mice. (**C**)Quantitative results for p-tau^S396^. Relative levels of p-tau^S396^ to total tau were plotted and were increased in *Dicer* cKO mice. No difference in relative p-tau^S396^ levels was found between minocyline- and saline-treated *Dicer* cKO mice. Tau5 antibody was used to detect levels of total tau. There was no difference between *Dicer* cKO and control mice. Raw Western blotting images for p-tau and total tau were shown in Supplementary Figure 7.

A previous study has shown that conditional deletion of Dicer in forebrain excitatory neurons results in neurodegeneration through affecting tau phosphorylation^37^. To examine whether tau phosphorylation is increased in this line of *Dicer* cKO and is affected by minocycline, we used antibodies specifically against tau phosphorylated at several epitopes (p-tau) to conduct Western analyses (Fig. 6A).

For p-tau^Thr205^ (Fig. 6B), compared to age-matched littermate controls, *Dicer* cKO mice showed a dramatic increase (control = 100% ± 8.6%, cKO = 284.5% ± 25.5%). However, minocycline did not affect p-tau^Thr205^ levels in cKO groups (284.5% ± 25.5% for “minocycline” vs 256.0% ± 57.0% for “saline”). ANOVA confirmed a significant genotype effect (p < 0.001) but no drug effect (p > 0.7). For p-tau^Ser396^ (Fig. 6C), *Dicer* cKO mice showed increased levels (control = 100% ± 8.0%, cKO = 156.7% ± 6.5%). We found that minocycline did not affect p-tau^Ser396^ levels in cKO groups (156.7% ± 6.5% for “minocycline” vs 151.9% ± 21.7% for “saline”). Levels of total tau were not changed in *Dicer* cKO mice and were not affected by minocycline (data not shown). Overall, the findings on tau phosphorylation in this line of *Dicer* cKOs are in general agreement with those reported previously^37^.

**Figure 7:**
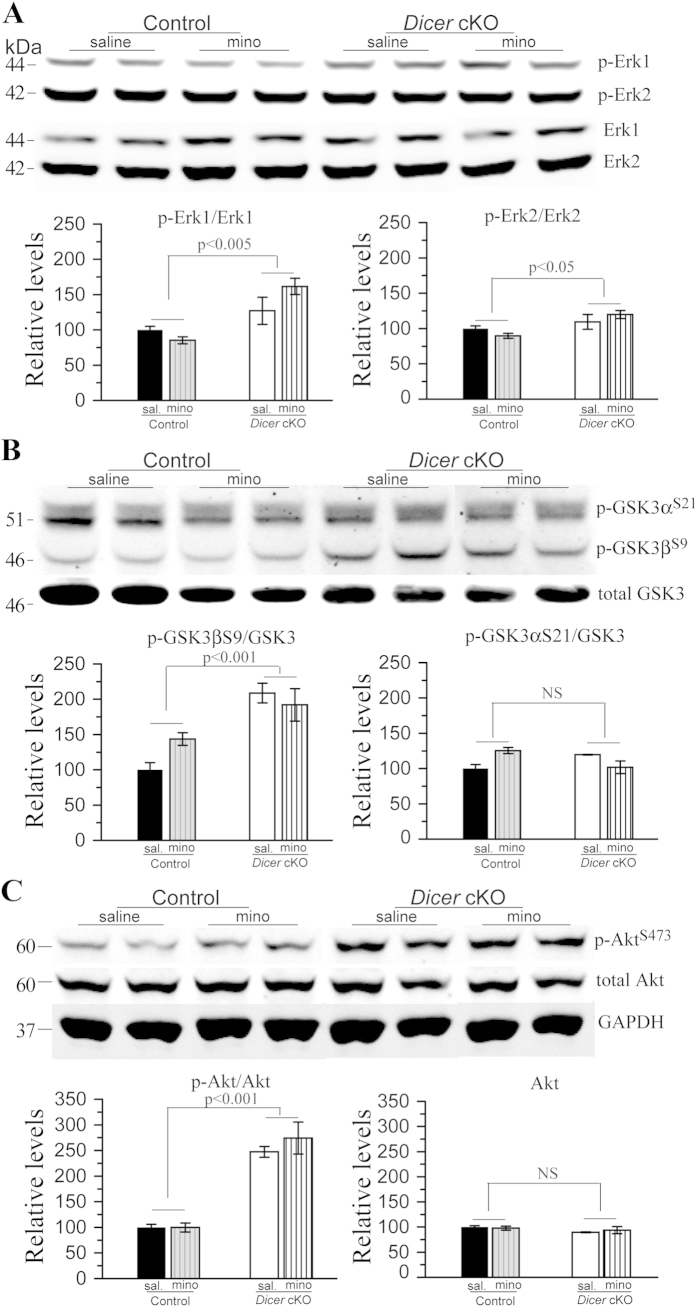
Minocycline did not affect activities of tau upstream kinases in *Dicer* cKO mice. (**A**) Western blotting for Erk1/2. Representative bands for p-Erk1/2 and total Erk1/2 were shown. For quantification analysis on Erk1, Relative levels of p-Erk1 to total Erk1 were significantly increased in *Dicer* cKO mice, as compared to control animals. But there was no difference between minocyline- and saline-treated *Dicer* cKO mice. For quantification analysis on Erk2, relative levels of p-Erk2 to total Erk2 were increased in *Dicer* cKO mice (p < 0.05). There was no difference between minocyline- and saline-treated *Dicer* cKO mice. (**B**) Western blotting for GSK3α and GSK3β. Representative bands for p-GSK3β^S9^/p-GSK3α^S21^ and total GSK3 were shown. For quantification analysis on GSK3β^S9^, relative levels of p-GSK3β^S9^ to total GSK3 were significantly increased in *Dicer* cKO mice, as compared to control animals. For quantification analysis on GSK3α^S21^, relative levels of p-GSK3α^S21^ to total GSK3 were not changed in *Dicer* cKO mice. (**C**)Western blotting for p-Akt and total Akt. Representative bands for p-Akt^S473^ and total Akt were shown. For quantification analysis, relative levels of p-Akt^S473^ to total Akt were significantly increased in *Dicer* cKO mice, as compared to control animals. There was no difference between minocyline- and saline-treated *Dicer* cKO mice. Raw Western blotting images for phosphorylated Erk1/2, GSK3α, GSK3β and Akt were shown in Supplementary Figures 8–10.

Dicer may regulate tau phosphorylation through affecting activities of several tau kinases including Erk1/2 and GSK3β^37^. We conducted Western blotting to examine levels of p-Erk1/2 (Fig. 7A). For p-Erk1, ANOVA revealed a main genotype effect (p < 0.005) but no drug effect (p > 0.4). For p-Erk2, ANOVA showed a weak main genotype effect (p < 0.05) but no drug effect (p > 0.9). These results suggest that activities of Erk were increased in *Dicer* cKO mice but were not affected by minocycline. We then conducted biochemical analyses to examine GSK3α/β. As shown in Fig. 7B, we observed a significant genotype effect on p-GSK3β^S9^, as revealed by ANOVA (p < 0.001), suggesting that GSK3β activities were decreased in *Dicer* cKO mice. In contrast, we did not find significant genotype effect on levels of p-GSK3α^S21^ (p > 0.6), suggesting unchanged activities of GSK3α (Fig. 7B). We further examined Akt, a major GSK3 kinase, by Western analysis (Fig. 7C). A highly significant genotype effect was observed on levels of p-Akt^473^ (p < 0.001), suggesting increased activities of Akt in *Dicer* cKO mice. However, we did not find significant drug effect on p-Akt^473^ levels (Fig. 7C: p > 0.2). Overall, these results suggest that minocycline did not alter activities of Erk, GSK3 and Akt.

## Discussion

It remained unknown whether minocycline is an effective drug to prevent or to stop neuron loss in neurodegenerative diseases. In this study, minocycline was used to treat a mouse model displaying age-dependent neuron loss, synaptic loss and apoptosis in the cortex. We have shown that the treatment of minocycline successfully reduced neuroinflammatory responses but failed to ameliorate neuron loss and apoptosis. We also reported that the treatment of minocycline did not inhibit tau hyperphosphorylation.

Dysregulation of miRNAs contributes to neurodegenerative diseases including AD^43^,^44^,^45^,^46^. Indeed, a number of miRNAs were down-regulated in sporadic AD^47^,^48^,^49^,^50^,^51^. The expression of β-amyloid cleavage enzyme 1 (BACE-1), one of the key enzymes to produce Aβ, is regulated by several miRNAs including miR-15a and miR-107^44^,^48^. Interestingly, age-related neurodegeneration shown in the cortex of the *Dicer* cKO mouse is caused by specific loss function of miR-15a but not global miRNAs, as miR-15a affects tau phosphorylation^37^. Since *Dicer* cKO mice also exhibit a wide range of AD-like pathology such as age-related synaptic loss, apoptosis, tau hyperphosphorylation, neuroinflammation and neurogenesis impairment^16^,^37^, it is an excellent animal model to test potential therapeutic candidates for neurodegenerative diseases^52^. Unlike the *Dicer* cKO published previously^37^, the line used in this study did not exhibit early death, and therefore can be conveniently used to test anti-neurodegeneration drugs. For example, it can be treated before and after neuron loss or neuroinflammation has started, and the drug effect can be evaluated at various post-neurodegenerative stages.

Multiple lines of evidence have demonstrated that minocycline successfully inhibits neuronal death in mouse models of AD with amyloid pathology^21^, ALS^22^, HD^23^ and PD^24^. Interestingly, uncontrolled and prolonged neuroinflammation is believed to be critical for neurodegeneration^17^. Here, we designed a prevention study in which minocycline was used to treat the mice prior to overt neuroinflammation. The selection of this starting point for the drug treatment is important. First, in a recent treatment study, minocycline was administered to a sheep model of ND at a stage where neuroinflammation had already started^15^. Neither neuron loss nor neuroinflammation was suppressed^15^. Second, no improvement on plaque pathology was observed when the drug was given to APP Tg mice at a post-neuroinflammation stage^20^. In this study, while successfully inhibiting inflammatory responses, minocycline failed to ameliorate neuron loss in *Dicer* cKO mice. The above findings suggest that inhibition of neuroinflammation is not sufficient enough to prevent neuron loss. However, since only one dose of minocycline was used to treat the mouse model (this study) or the sheep model^15^, we can not rule out the possibility that higher doses of the drug may show protective effects.

The exact cause of neurodegeneration remains largely unknown. Our and other studies suggest that mechanisms for neurodegeneration and neuroinflammation are likely not the same. Previously, we have shown that apoptotic cells were detected as early as 11 weeks of age^16^ at which no neuron loss displayed in the *Dicer* cKO model. Here, we reported that apoptotic cells were markedly increased (Fig. 5) in *Dicer* cKO mice at 4 months when dramatic neuron loss was observed (Fig. 3). Interestingly, significantly increased number of apoptotic cells has been commonly observed in several cell-type specific *Dicer* cKO mice^32^,^36^,^53^. Increased apoptosis was also detected in other neurodegenerative mouse models at a pre-neurodegeneration stage, and became more severe at later stages^14^,^54^,^55^,^56^. Since a big amount of apoptotic neurons directly account for the neuron loss, apoptosis likely plays a key role in initiating and driving neurodegeneration observed in the mouse models discussed above.

Hyperphosphorylated tau is generally believed to cause neuron death in AD and FTD^31^,^57^. Here, we observed age-related tau hyperphosphorylation in *Dicer* cKO mice, as evidenced by increased levels of p-tau (Fig. 6). The latter is likely caused by increased activities of Erk1 but not GSK3, as levels of an activated form of Erk1 but not GSK3α/β were increased (Fig. 7). The findings on p-tau are in agreement with those reported in the Hébert *et al.* (2010) study. In our study, minocycline seemed not to affect p-tau levels in *Dicer* cKO mice. In contrast, it was found that minocycline reduces p-tau levels and insoluble tau aggregates in a tau Tg mouse model of AD^30^. The discrepancy is likely due to different mouse models used. For example, tau hyperphosphorylation is caused by overexpression of human tau in tau Tg mice^30^,^31^ but by enhanced Erk1 activities in *Dicer* cKO mice. Moreover, whereas no changes in p-tau levels were found in minocycline-treated *Dicer* cKO mice, levels for activated forms of several tau kinases were also not altered by the drug.

In summary, we have demonstrated that minocycline effectively inhibited neuroinflammation but failed to suppress neuron loss in a neurodegenerative mouse model. Due to a wide range of protective effects in different mouse models of various brain diseases^21^,^22^,^23^,^24^,^25^,^26^,^28^,^30^,^31^, minocycline has been proposed as a potential therapeutic agent for the treatment of ND^18^,^19^. However, the findings in this study, along with the failure of a clinical trial of minocycline in treating ALS^58^, strongly suggest that minocycline may improve neuroinflammation-related symptoms but not necessarily delay neuron death in human neurodegenerative diseases. In order to make better clinical outcome for ND, minocycline needs to be used in combination with other therapies targeted at different pathological pathways.

## Methods

### Animals

Floxed *Dicer* mice (*Dicer*^*f/f*^) and *CaMKIIα-Cre* transgenic (Tg) mice were obtained from the Jackson Laboratory (Bar Harbor, ME, USA). To generate mature neuron-specific *Dicer* cKO mice, *Dicer*^*f/f*^ were first crossed with *CaMKIIα-Cre* to obtain *Dicer*^*f/+*^*;CaMKIIα-Cre*. The latter were bred to *Dicer*^*f/f*^ to get age-matched *Dicer*^*f/f*^(control) and *Dicer*^*f/f*^*;CaMKIIα-Cre* (*Dicer* cKO) for experiments. Mice were housed in an SPF room of the core animal facility of the MARC (Model Animal Research Center of Nanjing University). The room temperature kept at 25 °C constantly and the light-cycle is automatically controlled (12 hrs for light and 12 hrs for dark). Animals had free access to food and water. The genetic background of all the mice used in this study was C57BL/6. Mouse breeding was conducted under IACUC approved protocols at the MARC. All the experiments were performed in accordance with the Guide for the Care and Use of Laboratory Animals of the MARC at Nanjing University. Great effort was made to reduce the total number of mice used and to minimize their suffering. The total number of mice used in this study was as follows, 8 for controls with or without minocycline treatment, 6 for *Dicer* cKO mice with or without minocycline treatment.

### Minocycline treatment

Minocycline was purchased from Sangon Biotech (BBI MD0356). The concentration of minocycline for this study was 10 mg/kg, the same as used by the Friedlander group^22^,^23^,^59^. Minocycline was freshly prepared in each injection day. Mice received intraperitoneal injection of minocycline hydrochloride in saline (“minocycline” group) or saline alone (“saline” group) for 2 months. Mice were sacrificed 2 hrs after the final injection and brains were dissected. All the experimental protocols on mice were approved by the institutional committee of the MARC at Nanjing University.

### Immunohistochemistry

Mice were perfused with PBS^42^. The brain was dissected out and then fixed in 4% paraformaldehyde (PFA) overnight. After the fixation, the brain was washed using PBS for several times. Brains were dehydrated and then embedded in paraffin. Paraffin blocks were sectioned at the thickness of 10 μm. For IHC, saggital sections were deparaffinized, ethanol hydrated and then incubated with monoclonal antibodies against GFAP (1:1000; Sigma-Aldrich, Saint Louis, US), NeuN (1:500; Millipore, Billerica, US), MAP2 (1:500, Sigma-Aldrich), SVP38 (1:500, Sigma-Aldrich). The slides were rinsed with PBS for several times to wash out the first primary antibody, biotinylated goat anti-mouse IgG (vector,1:500) was used as the secondary antibody. Signals were amplified using the ABC (avidin-peroxidase complex) kit (Vector). After the reaction with DAB (Vector), sections were dehydrated by ethanol and xylene, and then mounted using neutral resin. For fluorescence immunostaining, the following secondary antibodies were used: Alexa Fluor 488 goat anti-mouse and Alexa Fluor 594 goat anti-mouse (Invitrogen). The dilution of the second antibody was 1:500. Sections were scanned using a Leica TCS SP5 laser confocal microscope.

### Tissue preparation

Mice cortices were dissected and homogenized in cold radio immunoprecipitation assay lysis buffer [consisting of the following (in mM): 20 mM Tris-HCl, pH 7.4, 150 mM NaCl, 1 mM EDTA, 1% NP-40, 0.5% sodium deoxycholate, and 0.1% SDS] containing protease and phosphatase inhibitors^60^. Lysates were cleared by centrifugation (14,000 rpm for 20 min).

### Immunoblotting

The same methods for Western blotting as those we described previously have been used in this study^14^,^16^,^42^,^60^. For each protein to be examined, e.g. GFAP, NeuN, Iba1 and IL6, cortical samples for all the mice were divided into 2 sets for gel-running (each set consists of 13-14 samples including 3-4 controls with saline, 3-5 controls with minocycline, 3 cKOs with saline and 3 cKOs with minocycline). Normalized volumes of samples (40 μg total protein) were resolved in 10% 15-well NuPAGE Bis-Tris gels (invitrogen), transferred to nitrocellulose membrane. After blocking with 5% (w/v) dry milk for 1 h, membranes were probed with primary antibodies overnight. The membrane was washed using TBS for three times, and then incubated with one of the Li-Cor IRDye infrared dye-coupled secondary antibodies, such as goat anti-rabbit IRdye800, goat anti-rabbit IRdye680, goat anti-mouse IRdye800 and goat anti-mouse IRdye680. Membranes were scanned using Odyssey Infrared Imaging System (Odyssey Image Studio by Li-Cor).

After scanning, the targetrd bands with correct molecular weight for each molecule in each image were processed by the Odyssey Image Studio for band intensity analysis, and data were then exported to Excel. The same membrane was then re-probed with primary antibodies against GAPDH (or β-actin) for analyses on intensities for GAPDH (or β-actin) as the internal control. For each protein in each lane, relative levels of one protein/molecule =band intensity of the targeted protein/band intensity of GAPDH (or β-actin). For p-tau, relative levels =p-tau band intensity/total tau band intensity. Relative values for different groups were then averaged for each group. The averaged value for the control group without minocycline was defined as the baseline (always 100% for the control group without minocycline). Levels for the other three groups were calculated relative to the control group without minocycline.

Primary antibodies used were as follows: anti-NeuN (1:500; Millipore), anti-GFAP (1:500; Sigma-Aldrich), anti-SVP38 (1:1000, Sigma-Aldrich). anti-IL6 (1:200; Cell Signaling, Danvers, US), anti-p-tau^Thr205^ (1:200; Invitrogen, Carlsbad, US), anti-p-tau^Ser396^ (1:200; Invitrogen), anti-p-tau^Thr231^ (1:500; Millipore), anti-tau5 (1:200; Millipore), anti-p-GSK3α^S21^/3β^S9^ (1:500; Cell Signaling), anti-p-Erk1/2 (1:500; Cell Signaling), anti-p-Akt^473^ (1:200; Thermo Fisher, Waltham, US), anti-Akt (1:1000; Cell Signaling), anti-GPADH (1:10,000; Sigma-Aldrich) and anti-β-actin (1:10,000, Sigma-Aldrich).

### TUNEL staining

The brain sections were blocked using 5% of goat serum for 30 min followed by the treatment of Fluorescein (Roche) at 37 °C for an hour^14^,^56^. The slides were then washed using TBS (tris-buffered saline) for three times. TUNEL staining was analyzed using a Leica TCS SP5 confocal laser scanning microscope. The total number of TUNEL+cells in the cortex and the hippocampus were counted using a stereological method^14^.

### Statistical analysis

Data were presented as the mean ± SEM. Two-way ANOVA was conducted to analyze genotype, drug or genotype×drug interaction effect. Two-tailed student’s t-test for pair-wise comparisons was performed post hoc to examine the difference between minocycline- and saline-treated groups. P < 0.05 (*) was considered statistically significant.

## Author Contributions

S.C., J.H., C.Z., C.X., L.W. and X.Z. performed experiments. S.C., J.H. and G.C. analyzed the data; H.Y., Z.Y. and Y.S. contributed unpublished reagents; G.C. and Z.Y. designed the research; G.C. wrote the manuscript.

## Additional Information

**How to cite this article**: Cheng, S. *et al.* Minocycline reduces neuroinflammation but does not ameliorate neuron loss in a mouse model of neurodegeneration. *Sci. Rep.* **5**, 10535; doi: 10.1038/srep10535 (2015).

## Supplementary Material

Supplementary Information
