# Supplementary material for: Minocycline reduces neuroinflammation but does not ameliorate neuron loss in a mouse model of neurodegeneration
Source: Sci Rep. 2015 May 22;5:10535. doi: 10.1038/srep10535 (PMC4441131; doi:10.1038/srep10535)
Supplement: Supplementary Information [file srep10535-s1.pdf]

Minocycline reduces neuroinflammation but does not ameliorate neuron loss in a mouse model of neurodegeneration

Shanshan Cheng[1,†], Jinxing Hou[2,†], Chen Zhang[1], Congyu Xu[1], Long Wang[1], Xiaoxia Zou[1], Huahong Yu[2], Yun Shi[1], Zhenyu Yin[3,*] and Guiquan Chen[1,*]

†These authors contribute equally to this work.

*Corresponding authors: Dr. Guiquan Chen (chenguiquan@nju.edu.cn)

or Dr. Zhenyu Yin (zhenyuyin68@163.com)

1. Model Animal Research Center，MOE Key Laboratory of Model Animal for Disease Study, Nanjing University, 12 Xuefu Road, Nanjing, Jiangsu Province, China, 210061.

2. School of Life Sciences, Nanchang University, Nanchang, Jiangxi Province, China, 330031.

3. Department of Geriatric, Nanjing Drum Tower Hospital, Nanjing University Medical School, 321 Zhongshan Avenue, Nanjing, Jiangsu Province, China, 210008.

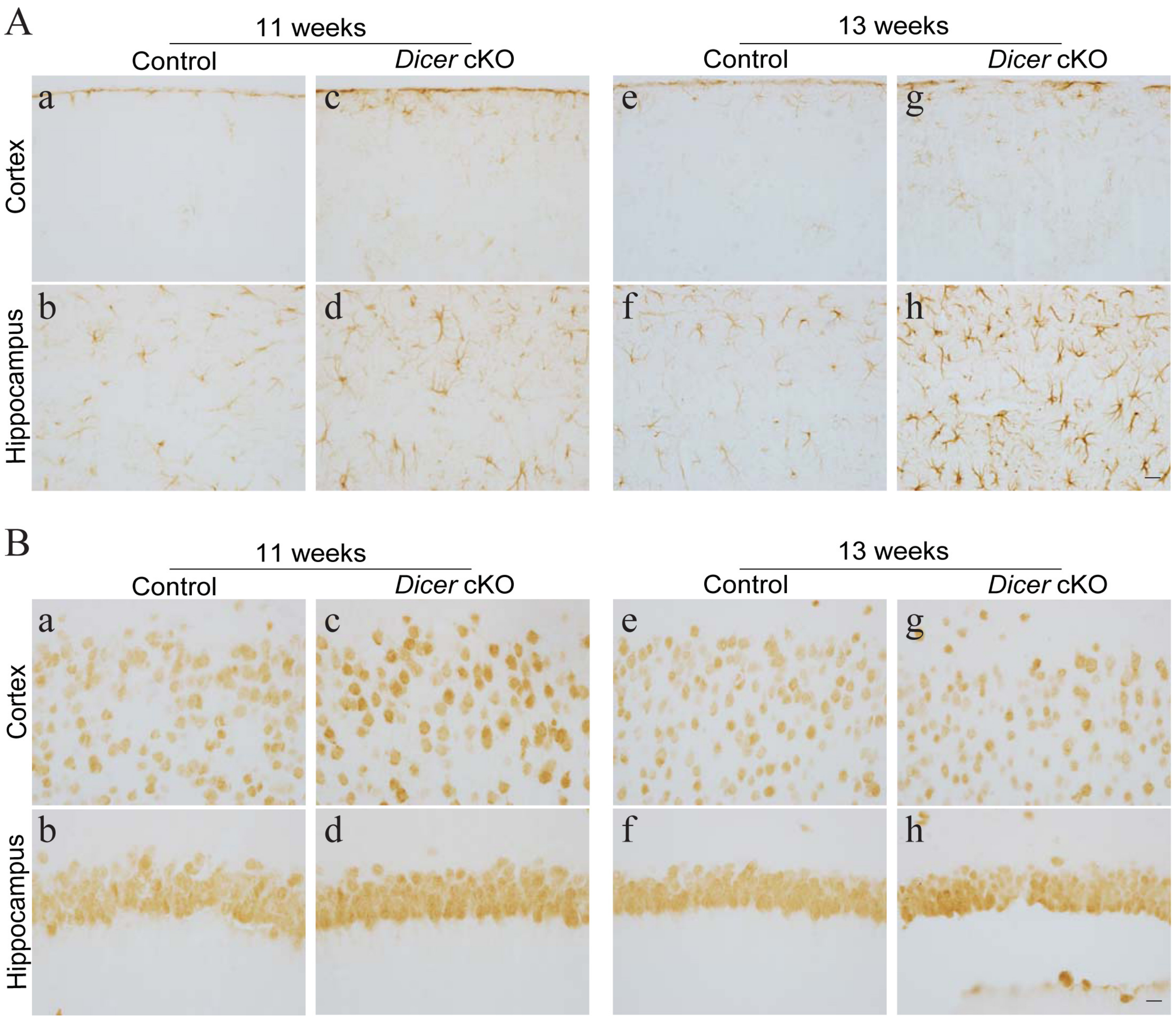

Supplementary Figure 1. Neuroinflammation but no neuron loss in 11 and 13 weeks old *Dicer* cKO mice. (A) Early astrocytosis in *Dicer* cKO mice. Immunohistochemistry of GFAP showed enhanced immuno-reactivity of GFAP in the cortex and the hippocampus of *Dicer* cKO mice at 11 (c,d) and 13 (g,h) weeks of age. (B) No significant neuron loss in young *Dicer* cKO mice. Immunohistochemistry of NeuN showed no detectable changes on immuno-reactivity of NeuN in the cortex and the hippocampus of *Dicer* cKO mice at 11 (c,d) and 13 (g,h) weeks of age. Scale bar=20µm.

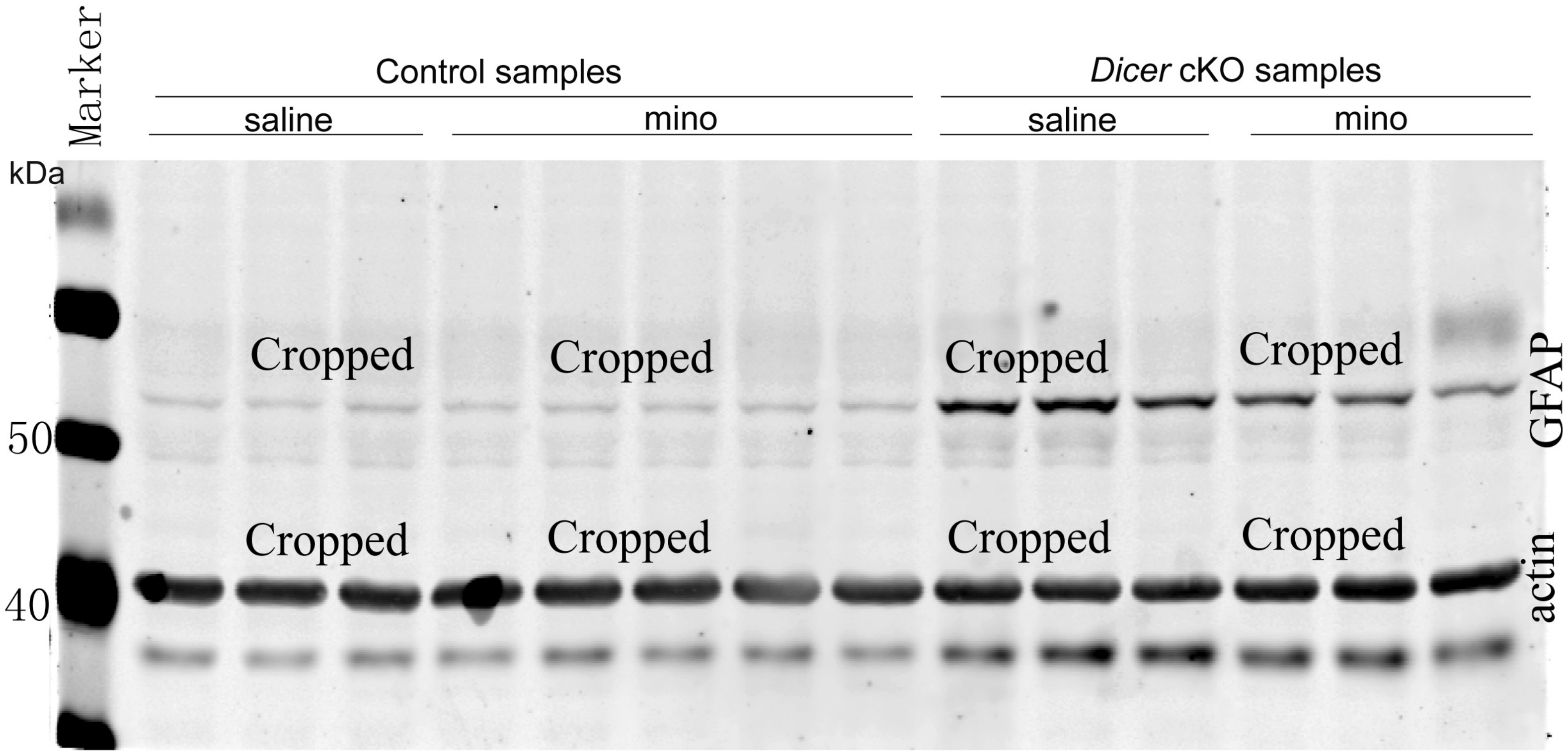

Supplementary Figure 2. Raw Western blotting for GFAP. 4 groups of cortical samples (40 µg total protein) were run using a 10% Bis-tris NuPAGE gel (invitrogen). The blot was probed with a GFAP antibody followed by a β-actin antibody. After incubations with secondary antibodies, The blot was scanned using the Licor Odyssey system. Two cropped bands for each group were marked and were used in Figure 2A.

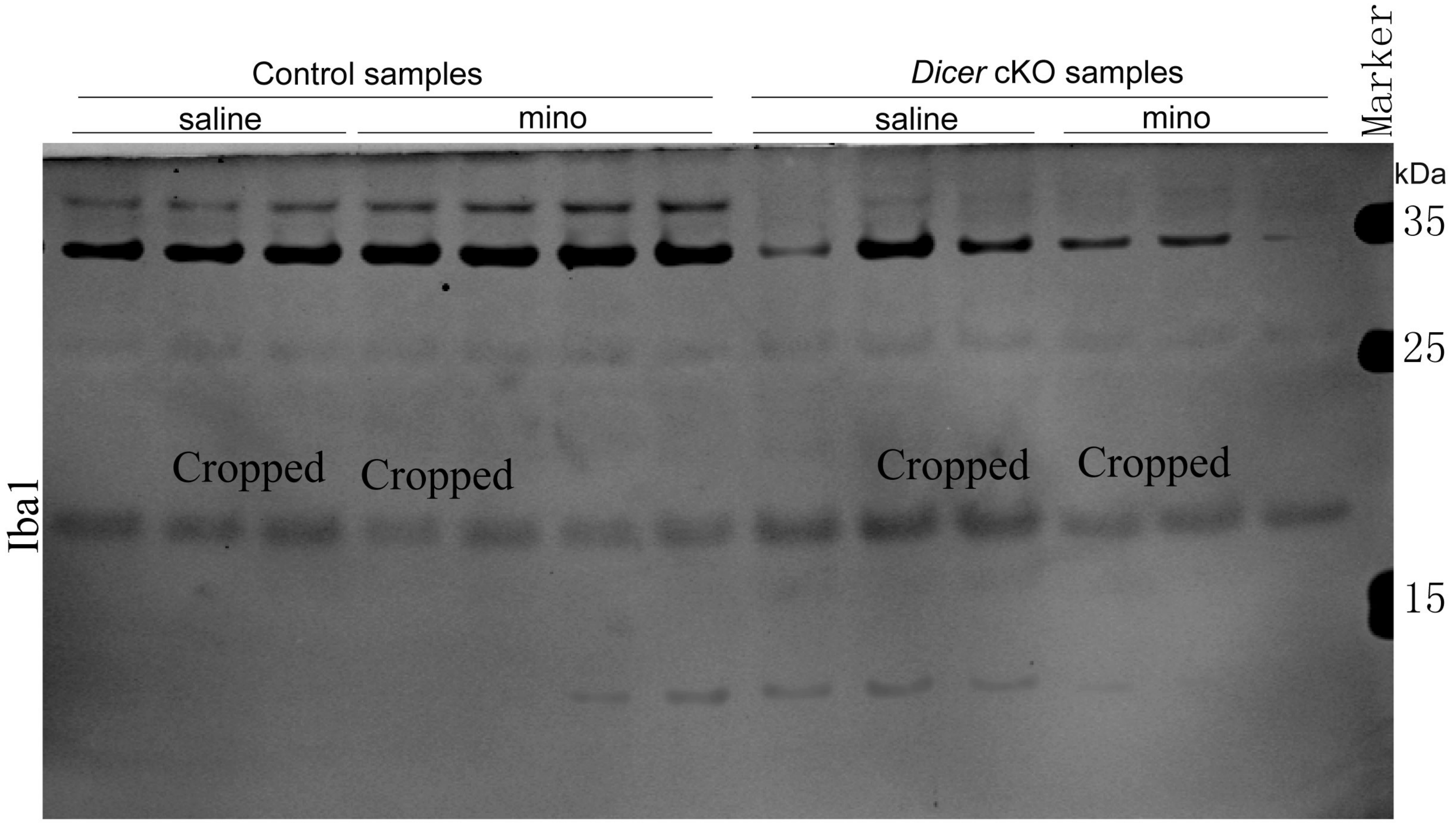

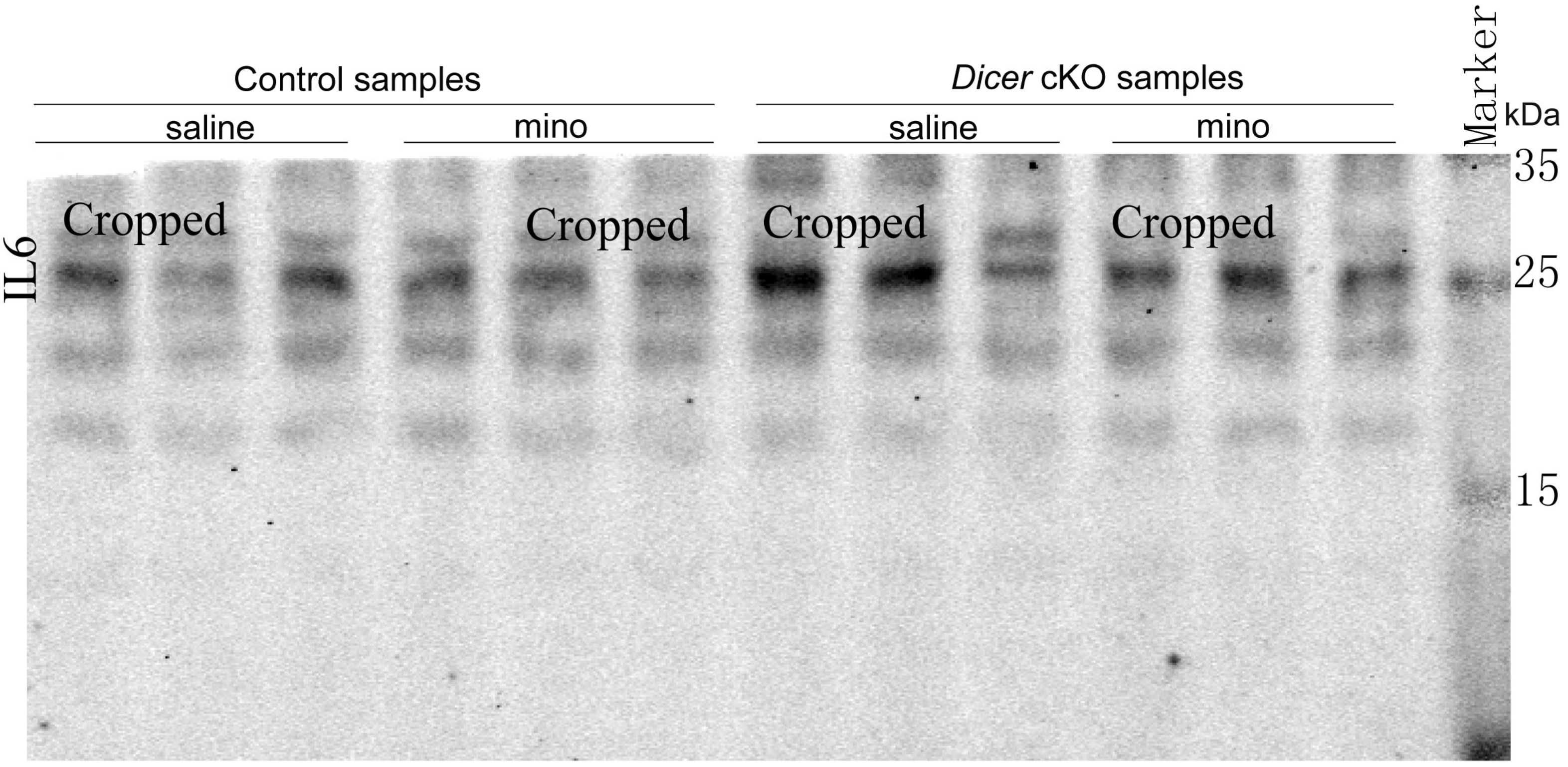

Supplementary Figure 3. Raw Western blotting for Iba1 (top) and IL6 (bottom). 4 groups of cortical samples (40 µg total protein) were run using a 10% Bis-tris NuPAGE gel (invitrogen). The blots were probed with Iba1 and IL6 antibody separately. After incubation with a secondary antibody, the blots were scanned using the Licor Odyssey system. Two cropped bands for each group were marked and were used in Figure 2A.

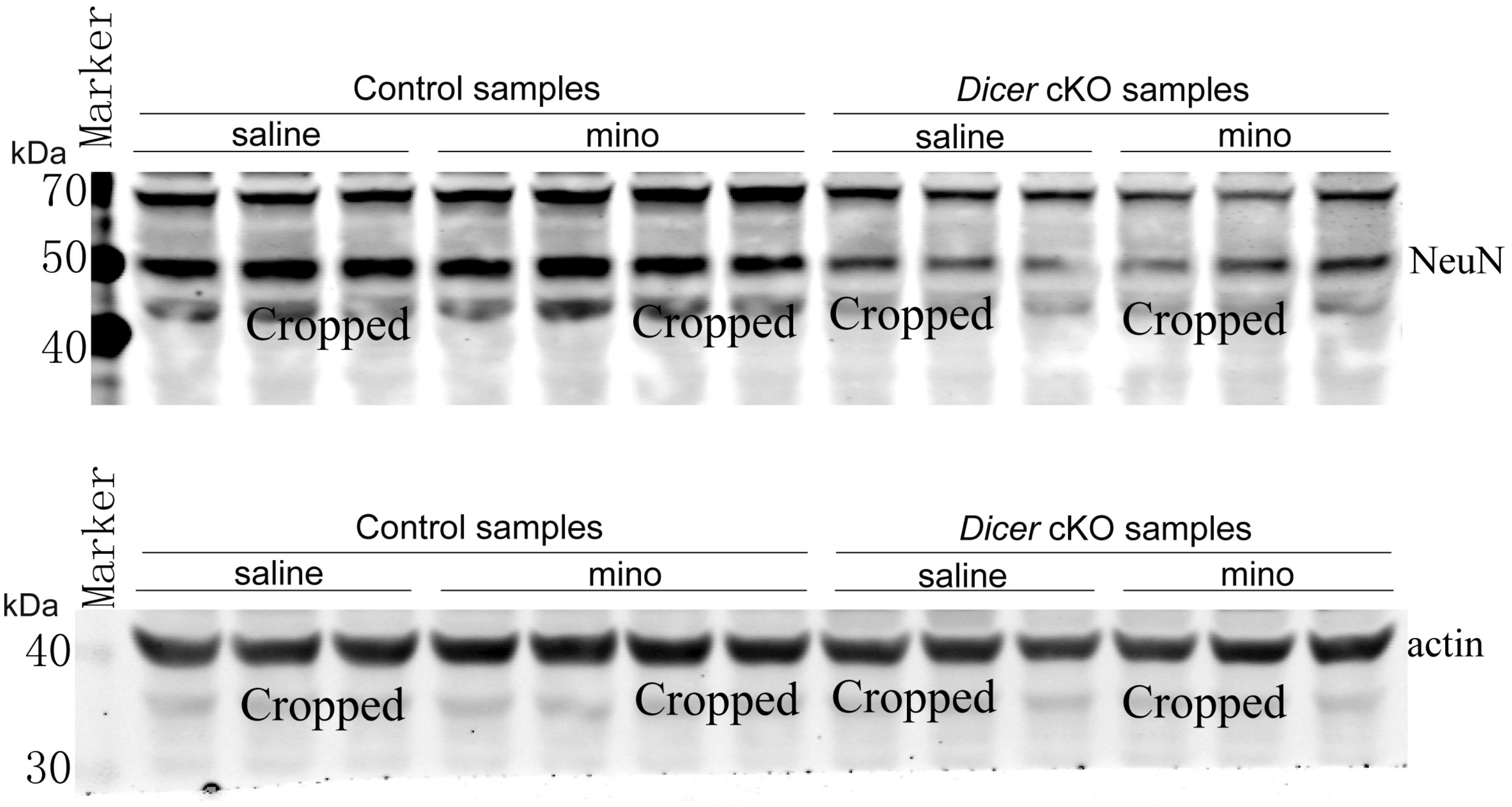

Supplementary Figure 4. Raw Western blotting for NeuN. 4 groups of cortical samples (40 µg total protein) were run using a 10% Bis-tris NuPAGE gel (invitrogen). The blot was probed with a NeuN antibody. After incubation with a secondary antibody, the blot was scanned using the Licor Odyssey system. Two cropped bands for each group were marked and were used in Figure 3A.

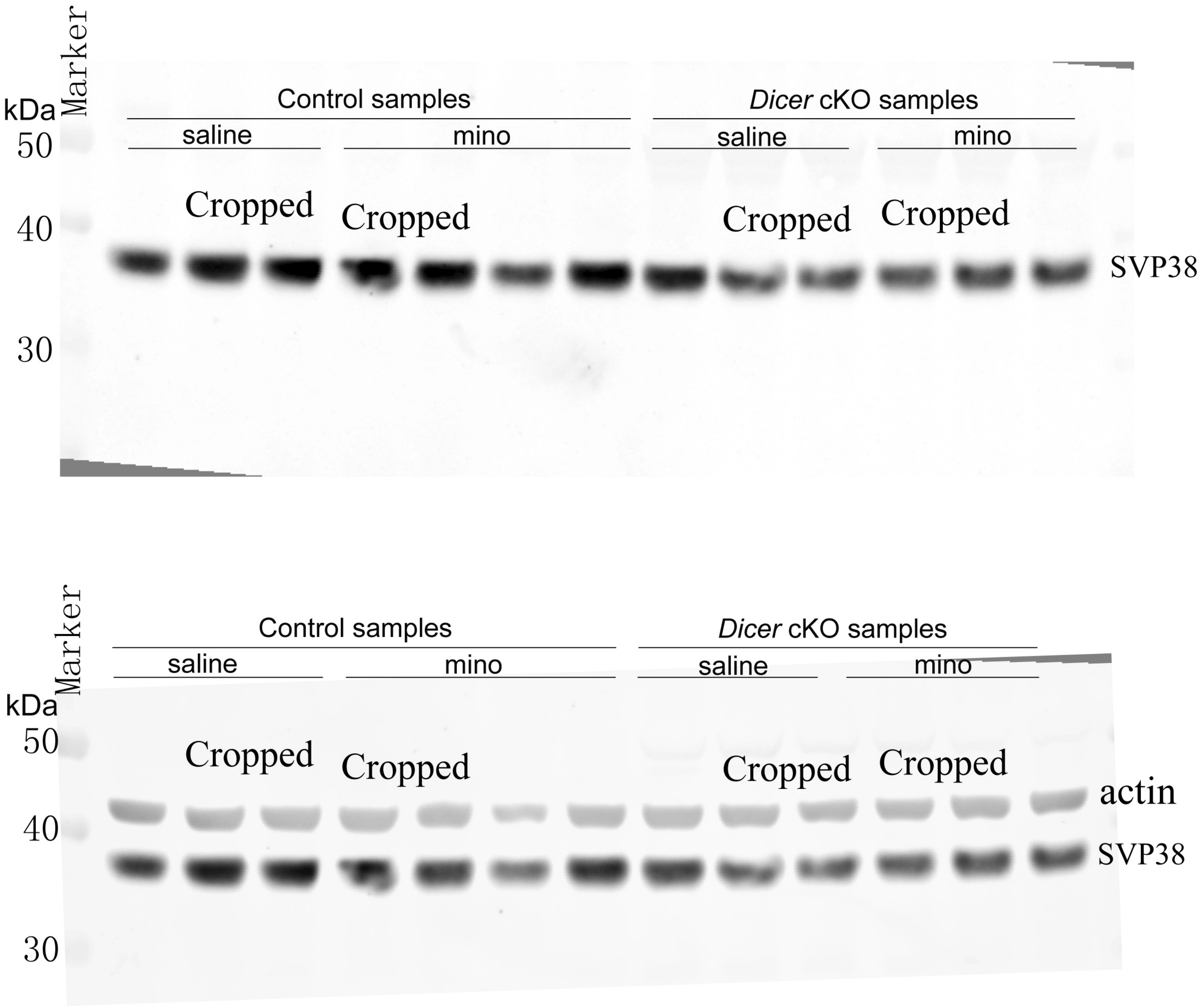

Supplementary Figure 5. Raw Western blotting for SVP38. 4 groups of cortical samples (40 µg total protein) were run using a 10% Bis-tris NuPAGE gel (invitrogen). The blot was first probed with an SVP38 antibody. After incubation with a secondary antibody, the blot was scanned using the Licor Odyssey system. The blot was then probed with a β-actin antibody. Two cropped bands for each group were marked and were used in Figure 4A.

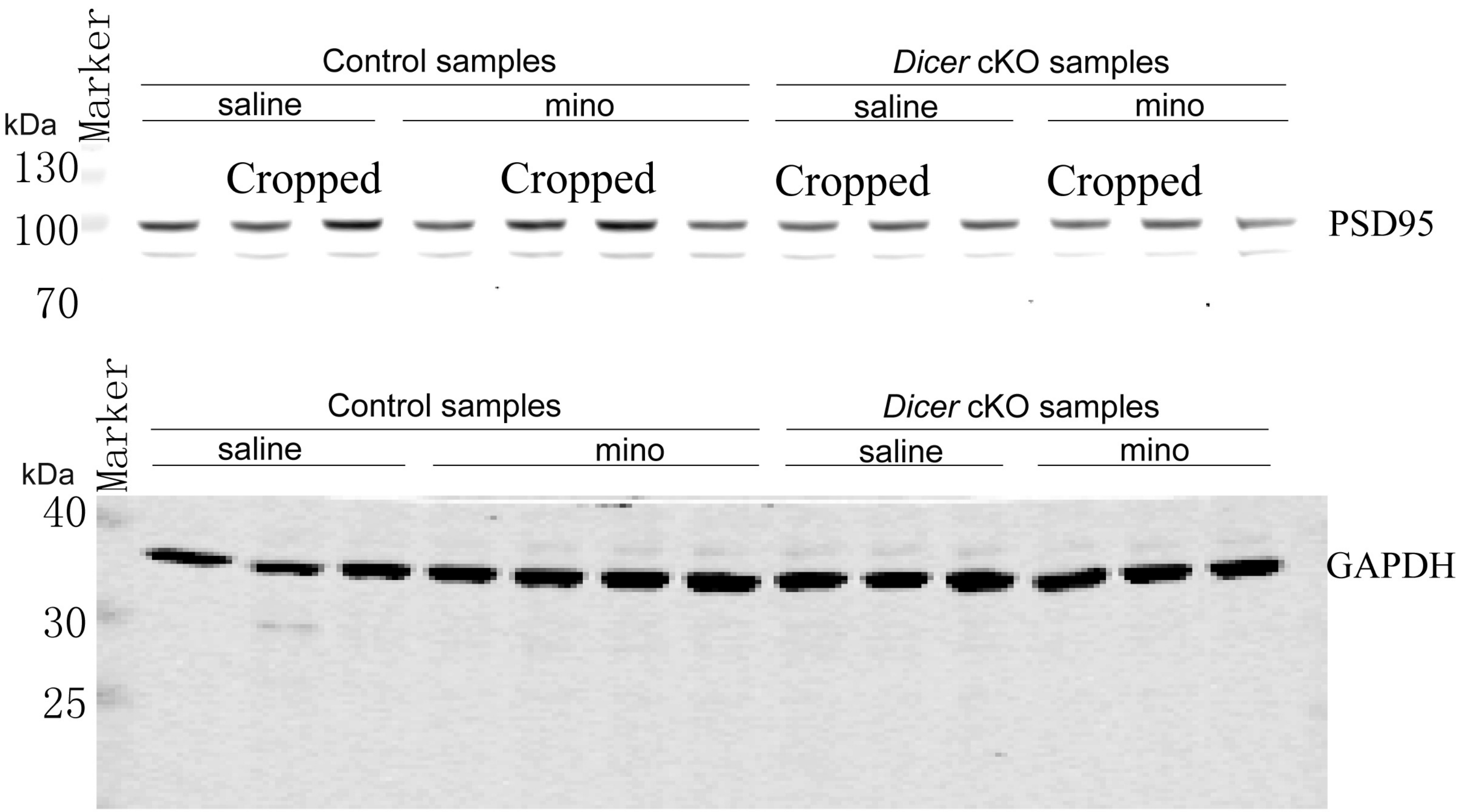

Supplementary Figure 6. Raw Western blotting for PSD95. 4 groups of cortical samples (40 µg total protein) were run using a 10% Bis-tris NuPAGE gel (invitrogen). The blot was probed with a PSD95 antibody. After incubation with a secondary antibody, the blot was scanned using the Licor Odyssey system. Two cropped bands for each group were marked and were used in Figure 4A.

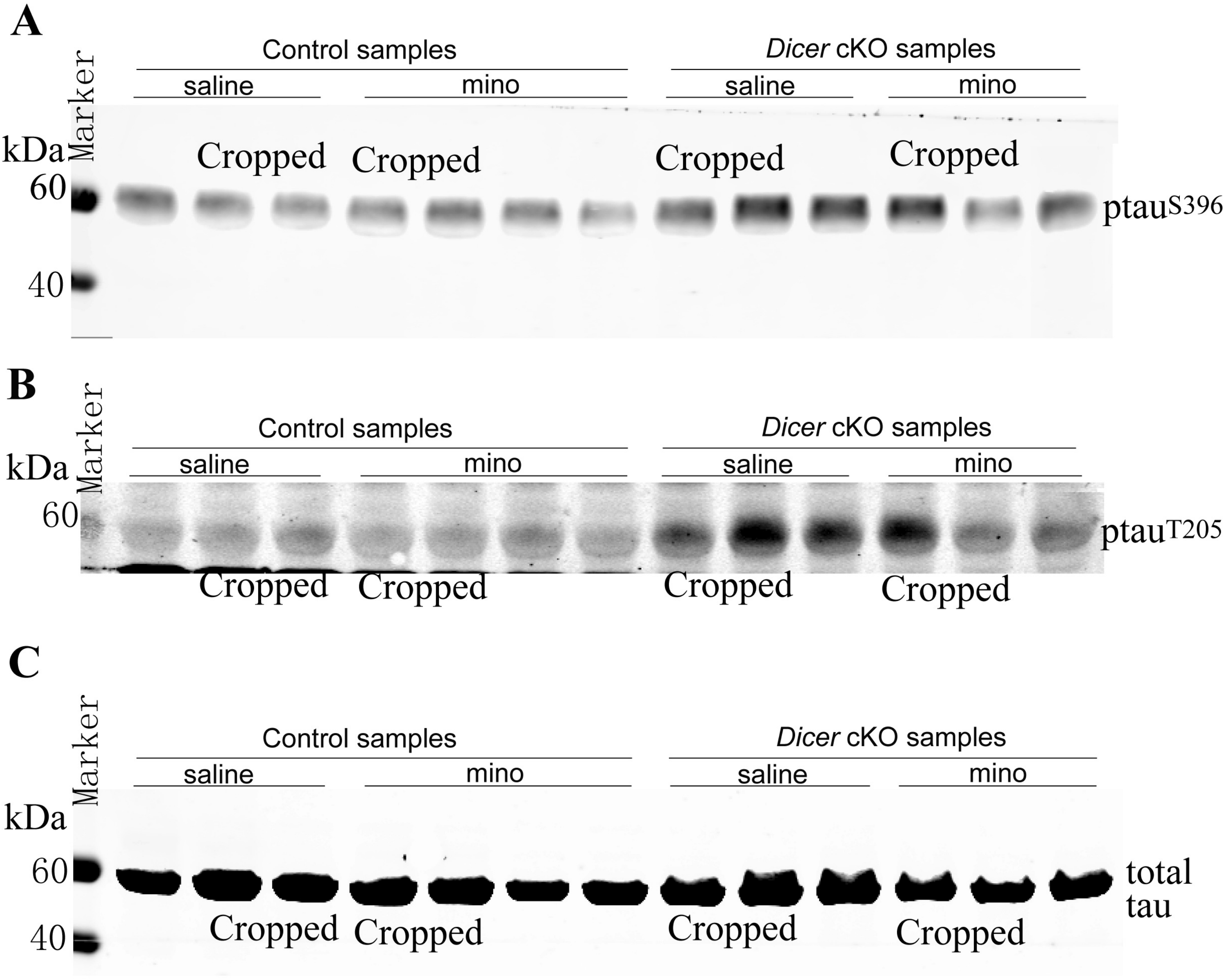

Supplementary Figure 7. Raw Western blotting for p-tau and total tau. 4 groups of cortical samples (40 µg total protein) were run using a 10% Bis-tris NuPAGE gel (invitrogen). These blots were probed with different p-tau antibodies separately. After incubations with secondary antibodies, the blots were scanned using the Licor Odyssey system. Two cropped bands for each group were marked and were used in Figure 6.

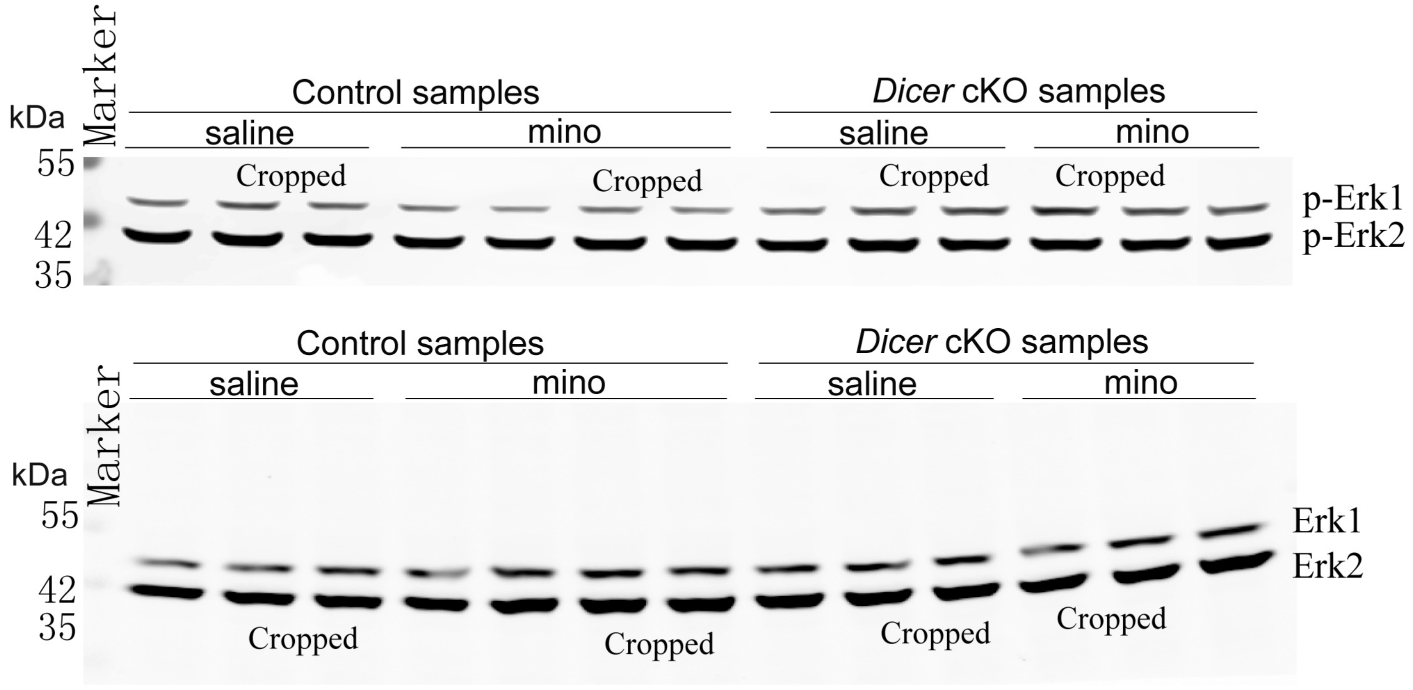

Supplementary Figure 8. Raw Western blotting for pErk1/2 and total Erk1/2. 4 groups of cortical samples (40 µg total protein) were run using a 10% Bis-tris NuPAGE gel (invitrogen). These blots were probed with pErk1/2 and total Erk1/2 antibodies separately. After incubations with secondary antibodies, the blots were scanned using the Licor Odyssey system. Two cropped bands for each group were marked and were used in Figure 7A.

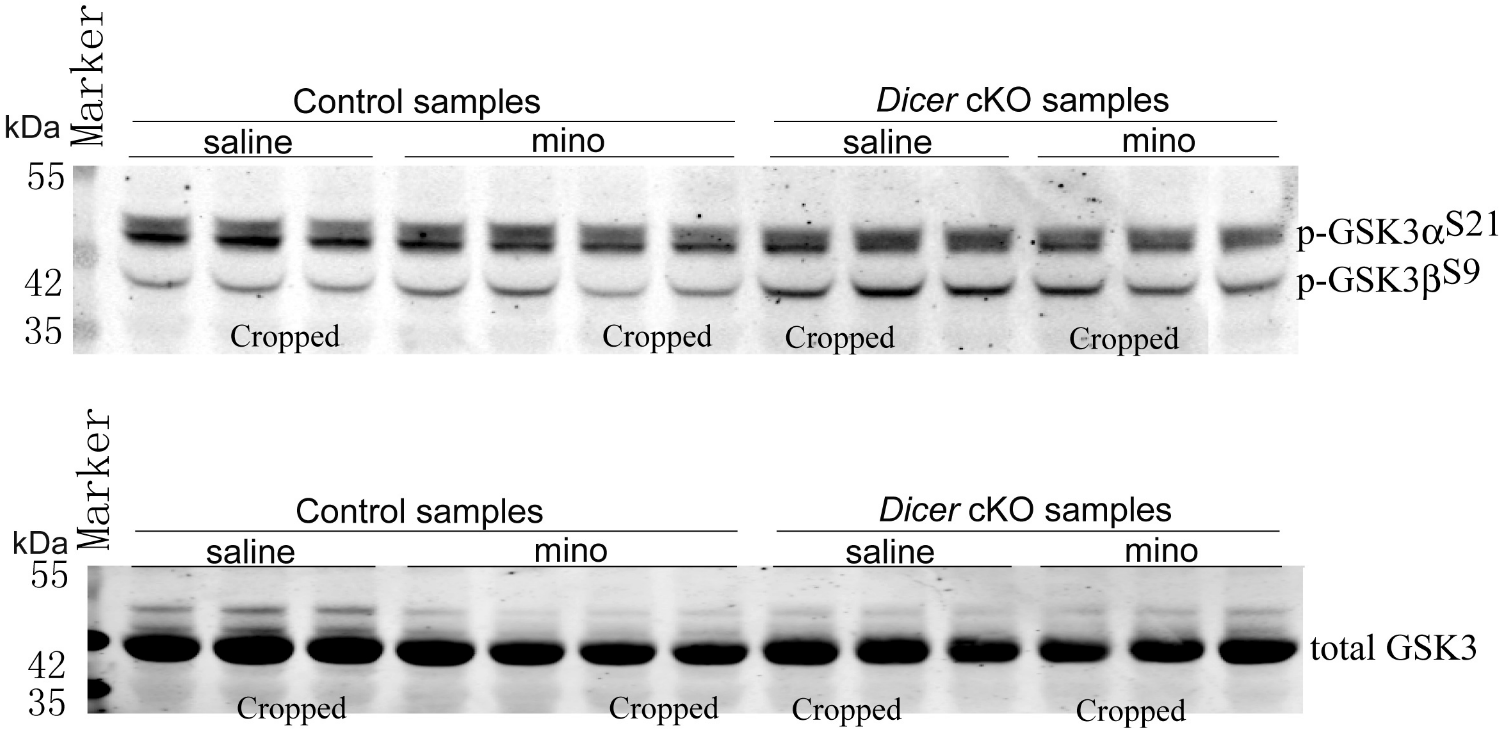

Supplementary Figure 9. Raw Western blotting for pGSK3α^{S21} and pGSK3β^{S9}. 4 groups of cortical samples (40 µg total protein) were run using a 10% Bis-tris NuPAGE gel (invitrogen). The blot was probed with an antibody against pGSK3α^{S21} and pGSK3β^{S9}. After incubation with a secondary antibody, the blot was scanned using the Licor Odyssey system. Two cropped bands for each group were marked and were used in Figure 7B.

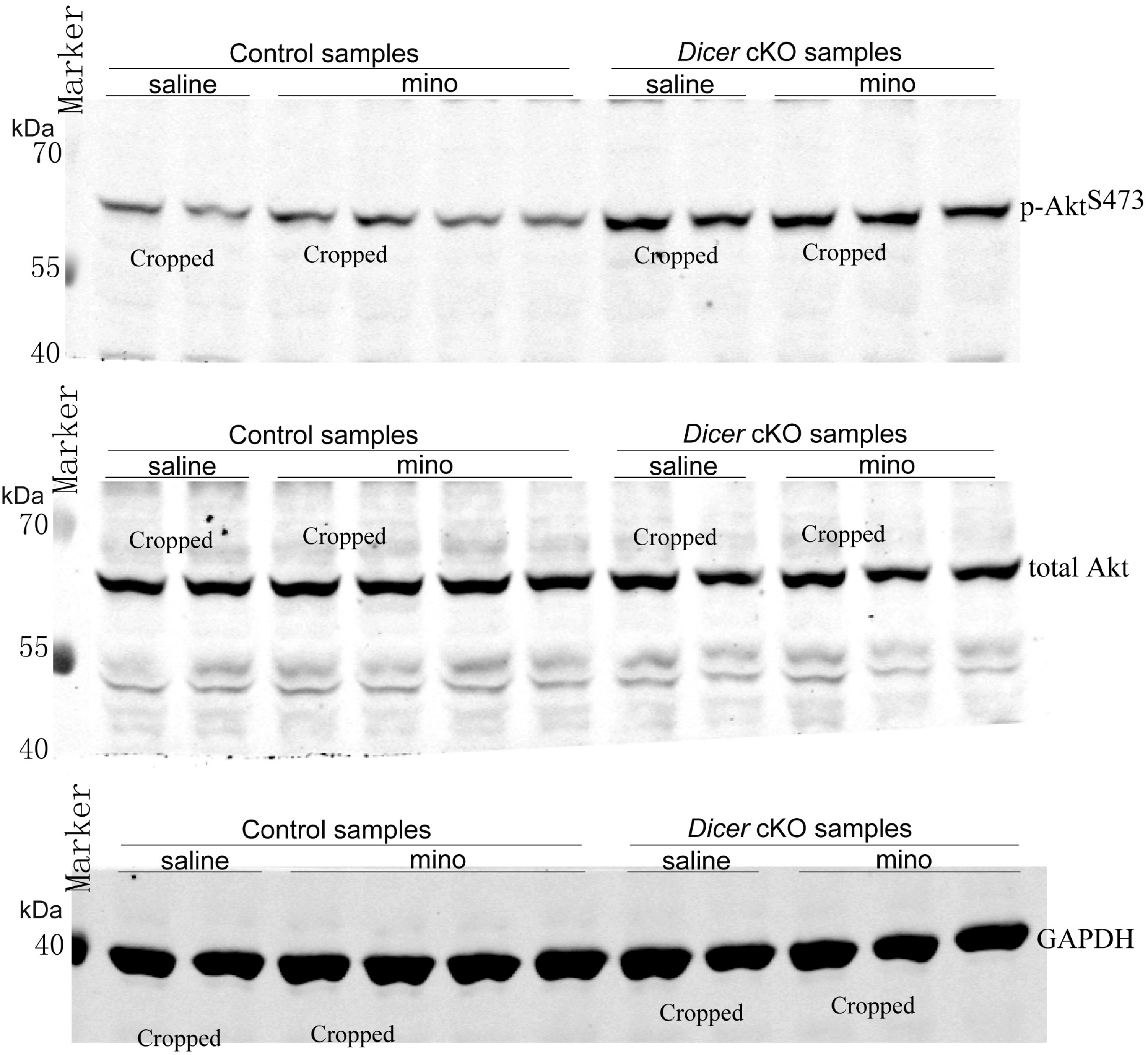

Supplementary Figure 10. Raw Western blotting for pAkt and total Akt. 4 groups of cortical samples (40 µg total protein) were run using a 10% Bis-tris NuPAGE gel (invitrogen). These blots were probed with pAkt and total Akt antibodies separately. After incubations with secondary antibodies, the blots were scanned using the Licor Odyssey system. Two cropped bands for each group were marked and were used in Figure 7C.
